# Metabolic and Lipid Biomarkers for Pathogenic Algae, Fungi, Cyanobacteria, Mycobacteria, Gram-Positive Bacteria, and Gram-Negative Bacteria

**DOI:** 10.3390/metabo14070378

**Published:** 2024-07-06

**Authors:** Paul L. Wood

**Affiliations:** Metabolomics Unit, College of Veterinary Medicine, Lincoln Memorial University, 6965 Cumberland Gap Parkway, Harrogate, TN 37752, USA; paul.wood@lmunet.edu

**Keywords:** microbe pathogenicity, algae, cyanobacteria, mycobacteria, bacteria, fungi, metabolomics, lipidomics, peptides

## Abstract

The utilization of metabolomics and lipidomics analytical platforms in the study of pathogenic microbes is slowly expanding. These research approaches will significantly contribute to the establishment of microbial metabolite and lipid databases of significant value to all researchers in microbiology. In this review, we present a high-level overview of some examples of biomarkers that can be used to detect the presence of microbes, monitor the expansion/decline of a microbe population, and add to our understanding of microbe biofilms and pathogenicity. In addition, increased knowledge of the metabolic functions of pathogenic microbes can contribute to our understanding of microbes that are utilized in diverse industrial applications. Our review focuses on lipids, secondary metabolites, and non-ribosomal peptides that can be monitored using electrospray ionization high-resolution mass spectrometry (ESI-HRMS).

## 1. Introduction

Metabolomics and the subfield of lipidomics are technologies that provide valuable data regarding microbial strain identification, metabolism, pathogenicity, drug-resistance, structural adaptations, and cell signaling [1,2]. Microbial lipids are significantly different from those of mammals, containing very long and very short fatty acyl chains attached to assorted headgroups. These headgroups include glycerol, sugars, fatty alcohols, amino acids, and peptides [3]. This is a review of these lipids and secondary metabolites unique to pathogenic microbes. We highlight a number of areas where high-resolution mass spectrometry has been utilized to assess the members of complex lipid or metabolite families. References to more detailed studies of individual families and to the mass spectral and tandem mass spectral data are provided.

It is important to raise a key issue at this point. There is only a limited number of studies in the literature regarding microbial lipidomics, and more research needs to be undertaken to increase our understanding of the biomarkers reviewed here. This is essential to define the mechanisms of microbial and polymicrobial biofilm formation. Biofilms are considered critical pathogenic factors in many acute and chronic infections and encompass a large variety of lipid molecules. The biomarkers in this review have all been verified by MS/MS and NMR analyses.

This review presents established lipid and metabolic biomarkers of pathogenic microbes. The utility of these biomarkers remains to be more fully explored. Our focus was on biomarkers that can be monitored in biofluids to assess microbial infections. Our review also focuses on electrospray high-resolution mass spectrometric (ESI-HRMS) data since this methodology provides both sensitivity and specificity to monitor even trace microbial infections. The need for HRMS is also stressed since lipid isobars can confound mass spectrometric data that are obtained with low resolution [3].

## 2. Pathogenic Algae, Cyanobacteria, and Fungi

*Prototheca* spp. and *Chlorella* spp. algae are opportunistic eukaryotes that enter damaged tissue surfaces and mucous membranes. Cyanobacteria are prokaryotes universally present in fresh and marine waters. Ingestion of these bacteria results in the absorption of a variety of toxins produced by cyanobacteria. The cell wall of algae is composed of polysaccharides and glycoproteins, while cyanobacteria lack a cell wall.

Pathogenic fungi are eukaryotes encompassing approximately 300 pathogenic species that possess a number of unique metabolic and lipid biomarkers. Fungi do not possess chloroplasts, and their cell wall is unique in that it includes a chitin–glucan polysaccharide complex.

### 2.1. Algal and Cyanobacterial Chloroplasts

**Figure 1:**
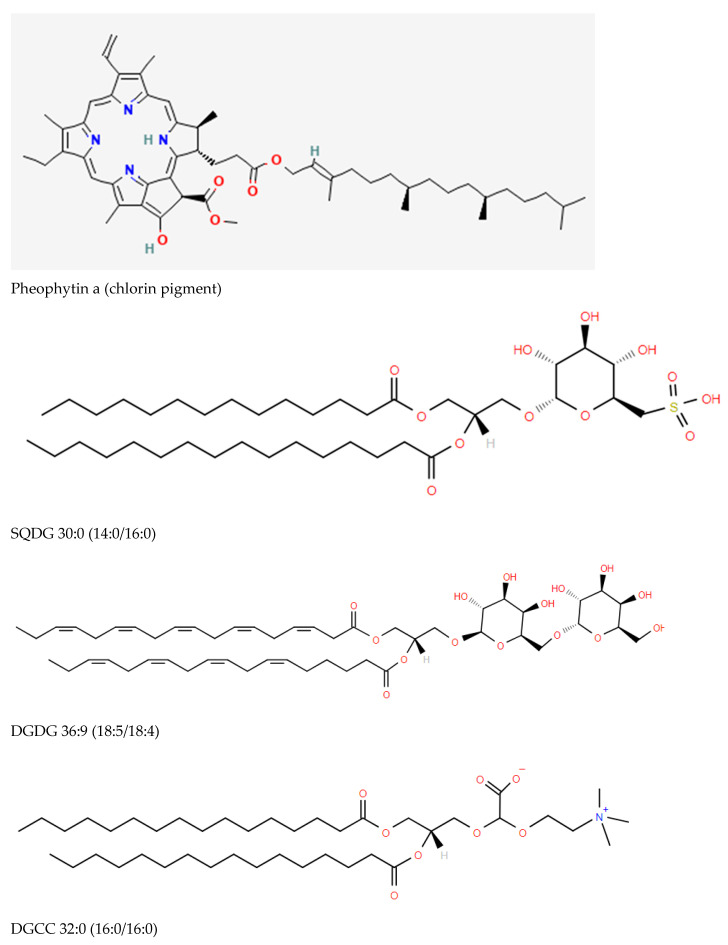
Structures of representative chloroplast biomarkers: chlorophylls (pheopytin a), sulfoquinovosyldiacylglycerols (SQDG; 1,2-diacyl-3-O-(6-sulfo-deoxy-D-glucosyl)-glycerol), digalactosyldiacylglycerol (DGDG), and diacylglyceryl carboxyhydroxymethylcholine (DGCC). DG-Hydroxymethyl-trimethyl-alanine (DGTAs) are isobars of DGCCs but have not been reported in bacteria.

Pathogenic algae and cyanobacteria both possess chloroplasts. Therefore, biomarkers of chloroplasts are useful indicators for these microbes. These include the following:

- Chlorophylls (e.g., chlorophyll a, pheophytin a) ([3,4,5]; Figure 1).
- Sulfoquinovosylmonoacylglycerols (SQMG) and sulfoquinovosyldiacylglycerols (SQDG) ([3,4,5]; Figure 1), which are sulfonolipids localized to the thylakoid membrane of chloroplasts functioning in the maintenance of photosystem II (PSII) [6,7,8]. Other possible functional roles include the regulation of DNA synthesis [9] and stimulation of glycosyltransferases involved in monohexosyldiacylglycerol (MHDG) and dihexosyldiacylglycerol (DHDG) synthesis [10].
- Glycerolipids, which includes MHDG and DHDG, are also localized to the thylakoid membrane of chloroplasts [3,4]. The hexosyl substituent can be glucose or galactose (Figure 1). However, these glycolipids are also essential lipids in the membranes of Gram-positive bacteria.
- Monoacylglyceryl carboxyhydroxymethylcholines (MGCCs) and diacylglyceryl carboxyhydroxymethylcholines (DGCCs) possess the same zwitterionic properties as choline and ethanolamine glycerophospholipids, making them available to substitute for these membrane glycerophospholipids [11].

### 2.2. Cyanobacterial and Fungal Non-Ribosomal Peptide Synthesis (NRPS)

**Figure 2:**
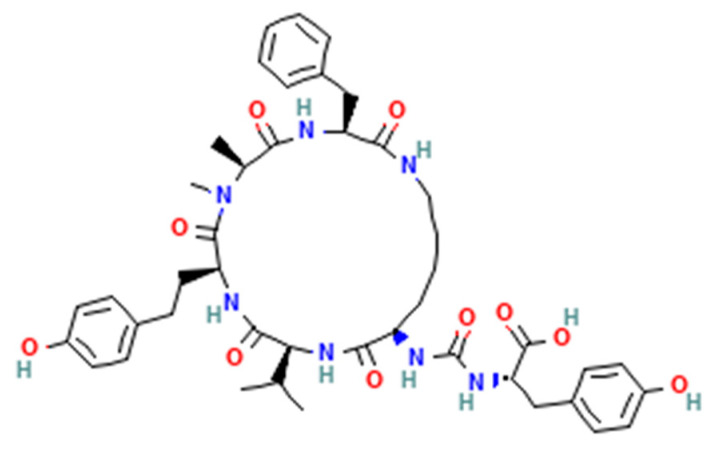
Structure of a representative non-ribosomal cyclic peptide of cyanobacteria. Anabaenopeptin A (Tyr-D-Lys-Val-Homotyrosine-MethylAlanine-Phe).

Bacteria and fungi possess non-ribosomal peptide synthetases (NRPSs), which are multi-modular enzyme complexes responsible for the biosynthesis of secondary metabolites. These include a number of linear or cyclic end-products [12,13,14]. In cyanobacteria, NRPSs generate a number of cyclic 5 amino acid peptide families (Table 1; Figure 2) with an exocyclic amino acid attached via a ureido linkage. The D-lysine in position 2 enables the cyclic structure via type III polyketide synthase. Amino acids can be modified by methylation, acetylation, and/or acylation via fatty acyl AMP ligases (FAALs). These secondary metabolites are potent enzyme inhibitors (proteases, carboxypeptidases, and phosphatases).

**Figure 3:**
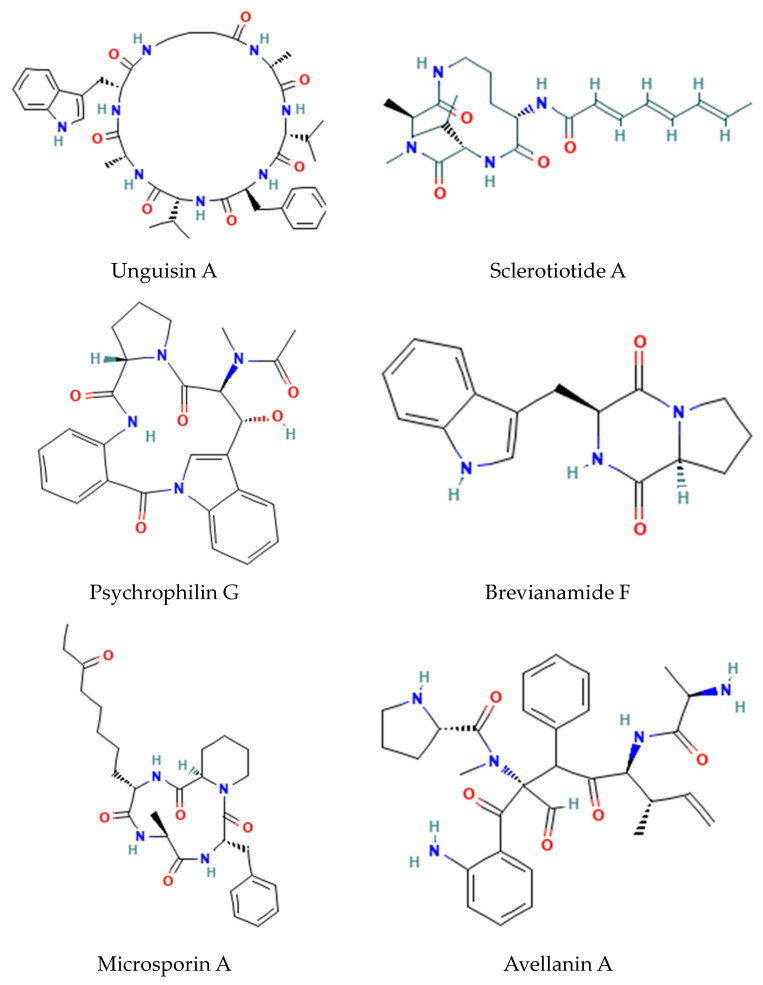
Structures of fungal antifungal di-, tri-, penta-, and hepta-peptides.

Fungal peptides are diverse in nature and unique from bacterial peptides but not as widespread as polyketides, alkaloids, and terpenoids. Examples of fungal antimicrobial cyclic peptides include (Figure 3) the following:

- Fungal GABA-containing cyclic heptapeptides: Unguisins [15];
- Fungal linear tri-peptides: Sclerotiotides, psychrophilins, and implicilliumtides [16];
- Fungal dipeptides: Brevianamides [17];
- Fungal cyclic tetrapeptide histone deacetylase inhibitors: Microsporins [16];
- Cyclic pentapeptides: Avellanins [16].

### 2.3. Cyanobacterial Fatty Acyl Organics

**Figure 4:**
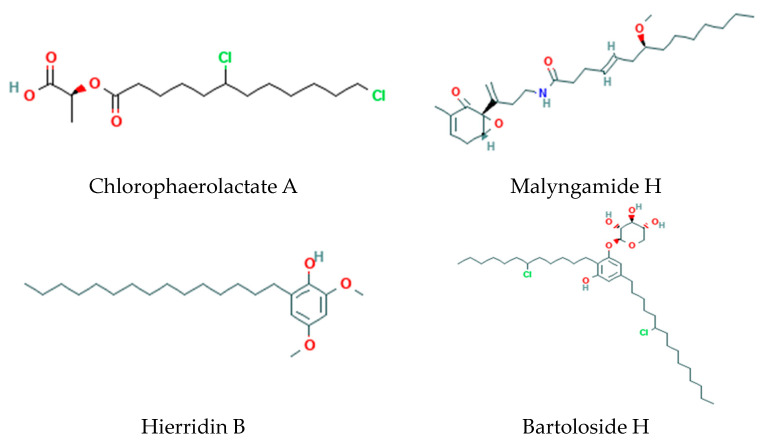
Structures of representative chlorosphaerolactylates, malyngamides, hierridins, and bartolosides.

Cyanobacteria possess polyketide synthases (PKSs) and acyltransferases involved in the biosynthesis of a wide diversity of secondary metabolites (Figure 4), which can be modified by methyltransferases, glycosyltransferases, and halogenases [18,19,20,21,22,23,24], as follows:

- Fatty acid esters:
  ○ Chlorosphaerolactylates;
  ○ Nocuolactylates;
  ○ The actions of lactylates remain to be elucidated since they only demonstrate weak antimicrobial activity [22].
- Monoterpinoids: Malyngamides, regulators of glucose transport, via Acyl(lipoyl) transferases.
- Acylphenols: Hierridins, voltage-dependent anion-selective channel 1 blockers, via type III PKS-catalyzed chain extension.
- Glyceryl-Chloro-Dialkylresorcinols: Bartolosides involving head-to-head condensation of two fatty acid-derived precursors via dialkylresorcinol/pyrone-forming and fatty acyl AMP ligase (FAALs) enzymes. The functions of these metabolites remain to be elucidated.

### 2.4. Cyanobacterial and Fungal Indole Alkaloids

**Figure 5:**
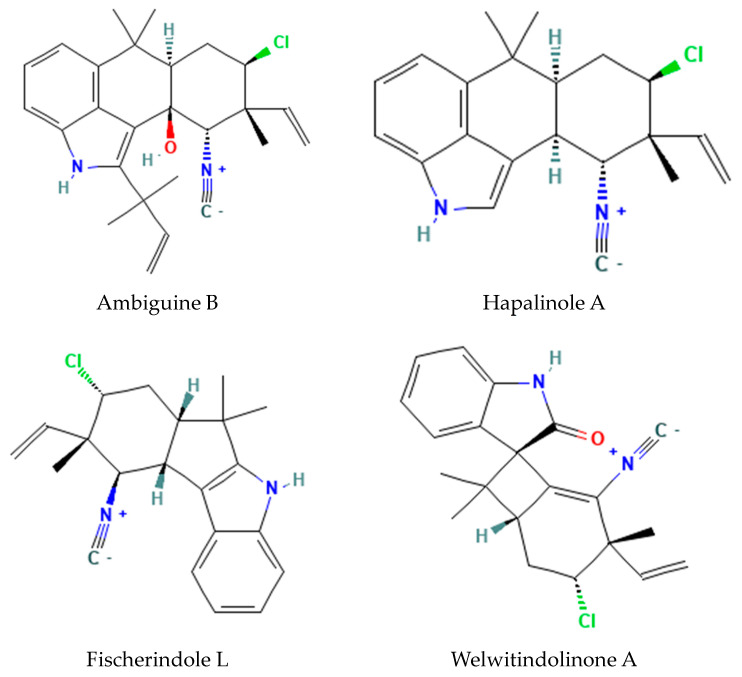
Structures of representative cyanobacterial indole alkaloids. These alkaloids are characterized by the reactive isonitrile functional group in all family members.

Cyanobacteria produce a variety of complex indole alkaloid families [19,20,21]. Several examples are presented in Table 2 along with their biological activities. These alkaloid families are characterized by reactive isonitrile functional groups. Most, but not all, family members are also halogenated with a chlorine substitution (Figure 5).

**Figure 6:**
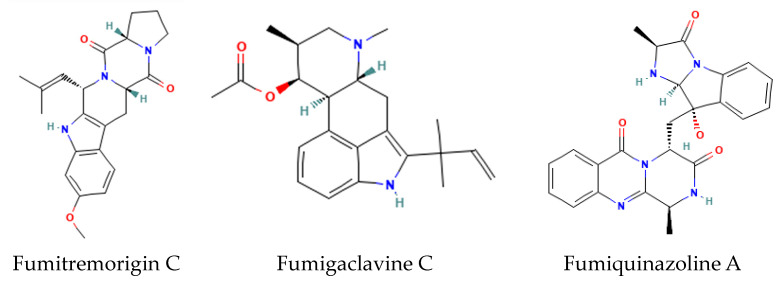
Structures of representative fungal indole alkaloid derivatives.

**Figure 7:**
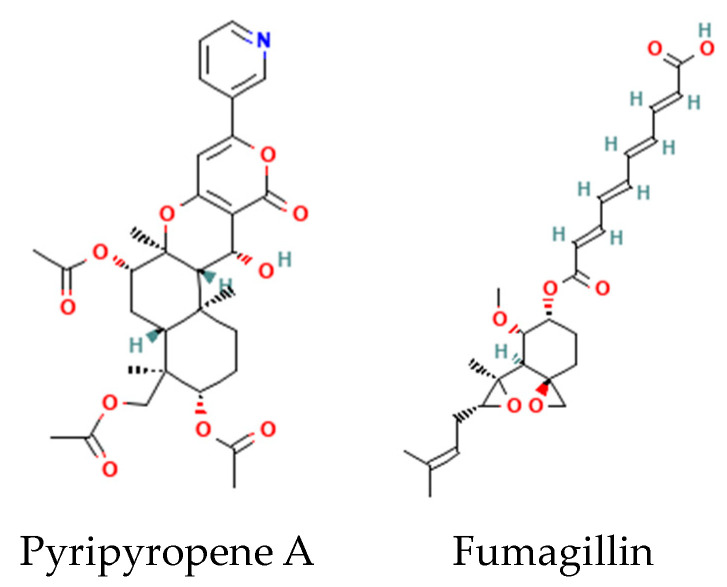
Structures of fungal sesquiterpenoid pyripyropene A and the complex isoprenoid fumagillin.

Fungal indole alkaloids are mycotoxins with complex ring extensions of the core indole nucleus. Examples include the fumitremorgins, fumigaclavines, and fumiquinazolines of *Aspergillus* sp. (Figure 6). Fungi produce a diverse array of other complex alkaloids (Figure 7). Several examples from *Aspergillus* sp. include sesquiterpenoid pyripyropenes and the complex isoprenoid fumagillin [25].

**Table 2:** Indole alkaloid families of cyanobacteria and fungi [26,27,28,29].

Cyanobacteria Indole Family	Biological Actions
Fischerindoles	Antibacterial
Hapalindoles	Antibacterial and antialgal
Welwitindolinones	Involved in multi-drug resistance development
Ambiguines	Fungicidal
**Fungal Indole Family**	**Biological Actions**
Apochalasins	Antibacterial
Fumitremorgins	Neurotoxic

The biosynthesis of alkaloids in cyanobacteria and fungi utilizes alkaloid synthesis gene clusters to provide the sequential enzyme activities. An example is the synthesis of fumigaclavine C (Figure 6), which requires a reductase followed by a P450 monooxygenase, followed by an acetyl transferase, and ultimately a prenyltransferase to generate the end-product, which is a virulence factor in *Aspergillus fumigatus* [26].

### 2.5. Carotenoids

**Figure 8:**
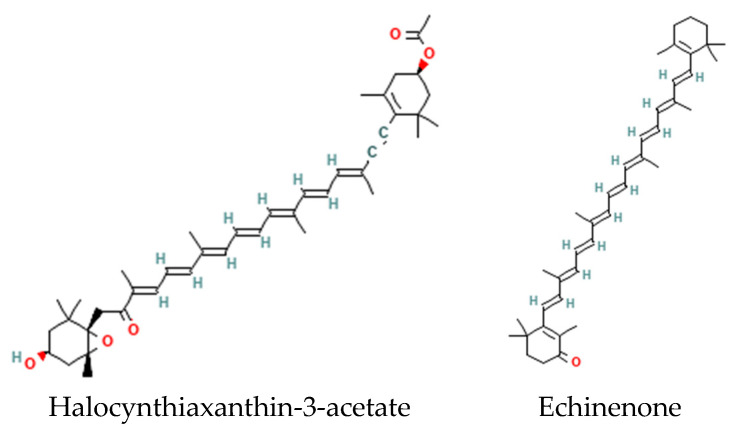
Structures of carotenoids halocynthiaxanthin-3-acetate (algae) and echinenone (cyanobacteria).

Algae and cyanobacteria contain high levels of several carotenoids not present in bacteria or fungi. The biosynthesis of carotenoids utilizes geranylgeranyl pyrophosphate as a precursor [27], generating halocynthiaxanthin-3-acetate and phoenicoxanthin in algae and echinenone in cyanobacteria (Figure 8).

### 2.6. Fungal Ergosterol

**Figure 9:**
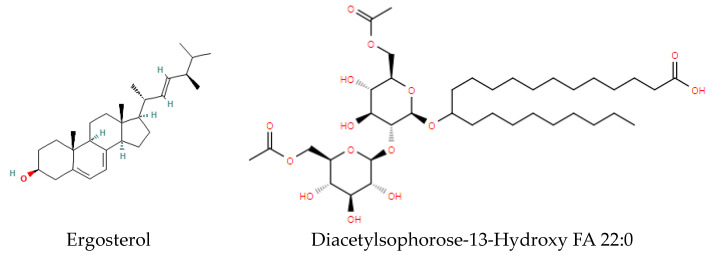
Structures of ergosterol and a representative sophorolipid.

Bacteria and fungi synthesize a wide variety of sterols. Fungi are unique in that the major membrane steroid is ergosterol (Figure 9), not cholesterol, representing a lipid biomarker for fungi [28].

### 2.7. Fungal Surfactant Glycolipids

Sophorolipids (Figure 9) are unique fungal lipid biomarkers but are restricted to non-pathogenic species [29]. Sophorose, acetyl-sophorose, or diacetyl-sophorose are the glyco substituents of a hydroxy fatty acid (Figure 9).

### 2.8. Fungal Glycosylinositol-Phosphorylceramides (GIPCs)

**Figure 10:**
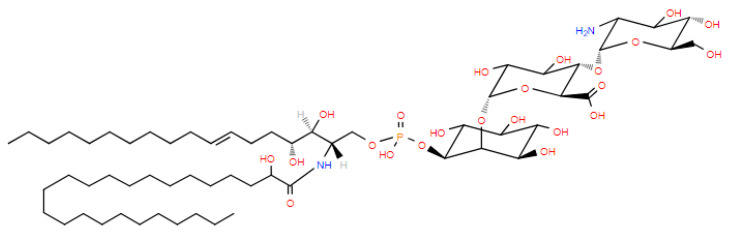
Structure of a GIPC (t18:1/h24:0).

GIPCs are membrane lipids with a glucuronic acid–inositol–phosphate substituent on a ceramide (Figure 10). These unique lipids are critical in the infection of a host. In filamentous fungi (e.g., *Aspergillus fumigatus*), these lipids are involved in adhesion, signal transduction, and modulation of the host immune response [30].

## 3. Mycobacteria

### 3.1. Mycolic Acids

**Figure 11:**
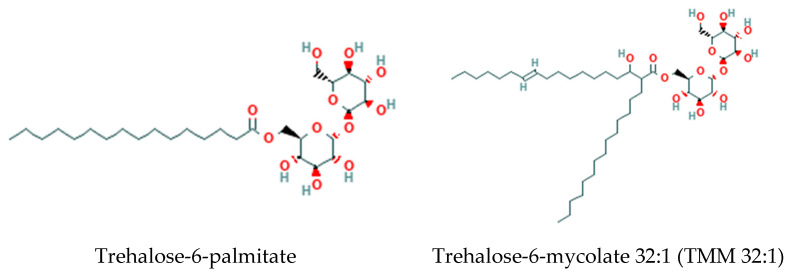
Structures of acyl trehalose lipids in mycobacteria.

The cell wall of *mycobacteria* is extremely complex, with the cytoplasm contained within a plasma membrane (lipid bilayer). The subsequent layers include the periplasm, the peptidoglycan layer, the arabinogalactan layer, and the outermost mycomembrane which is unique to Gram-positive mycobacteria (e.g., *Mycobacteria* spp. and *Salmonella* spp.). Lipids of the mycomembrane that can be monitored in free forms include mycolic acids, trehalose/glucose monomycolates (Figure 11), and dimycocerosates. The inner leaflet of the outer mycomembrane of mycobacteria contains high levels of mycolic acids [31,32,33]. These fatty acids are composed of a very long chain with a β-hydroxy substituent and an α-alkyl side-chain. The long-chain region can also contain cyclopropane rings, as well as methoxy or keto substituents. Another lipid family unique to *mycobacteria* is glycopeptidolipids (GPLs; Section 3.3), which are inserted at the cytoplasmic surface of the inner plasma membrane and the outer mycomembrane.

### 3.2. Trehalose Lipids

Disaccharide trehalose is synthesized de novo in mycobacteria and is used in the glycosylation of lipids [31,32]. These include acyltrehaloses, diacyltrehaloses, polyacyltrehaloses, trehalose mycolates, and trehalose dimycolates, which reside in the mycomembrane (Figure 11).

### 3.3. Glycopeptidolipids (GPLs)

**Figure 12:**
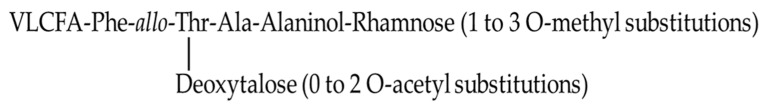
General outline of the structure of GPLs. Variants include replacement of the Phe by serine, valine, or leucine. VLCFA: very-long-chain fatty acids, which are 3-hydroxy or 3-methoxy. Amino acids are D isomers.

GPLs are complex hybrid molecules [33,34]. The peptidolipid scaffold consists of a 3-hydroxy or 3-methoxy fatty acid of 28 to 38 carbons linked to a tripeptide–aminoalcohol (alaninol) core (Figure 12). The tripeptide core consists of D-Phe-*allo*-Thr-D-Ala. Glycosylation involves a 6-deoxy-α-L-talose linked to D-*allo*-Thr and α-L-rhamnose linked to alaninol. Talose also can be mono- or di-acetylated, while rhamnose can be mono-, di-, or tri-methylated, adding to the complexity and diversity of the GPL family. GPLs at the external surface of the mycomembrane are thought to be involved in both bacterial sliding behavior and in the formation of invasive biofilms.

## 4. Gram-Positive Bacteria

### 4.1. Lipoteichoic Acids (LTAs)

**Figure 13:**
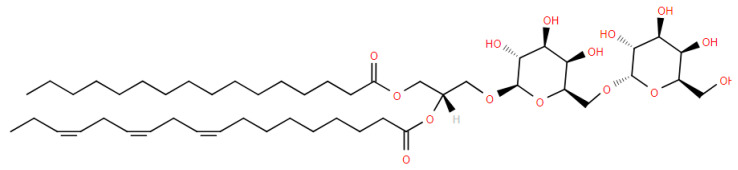
Structure of DHDG 34:3 (16:0/18:3). The H in this case is galactose. In the case of LTAPs, the GroP is attached to the terminal galactose residue.

Gram-positive bacteria possess a cytoplasmic membrane and a multilaminar cell wall [1]. Between the cell membrane and cell wall is a heteropolysaccharide meshwork of peptidoglycans and arabinogalactans. Teichoic and lipoteichoic acids anchor to peptidoglycans in the cell wall and, as such, are lipids unique to Gram-positive bacteria, providing a strong negative charge to the cell wall [2]. However, teichoic and lipoteichoic acids are large molecular weight polymers not amenable to simple extraction. Therefore, precursors to LTAs are monitored as biomarkers in Gram-positive bacteria. The specific lipids include dihexosyl diacylglycerols (DHDGs; Figure 13) and DHDG-glycerol phosphate (DHDG-GroP), with phosphoglycerol attached at the 6-hydroxy group of the terminal hexose, also termed an LTA primer (LTAP). LTAP can also be modified by mono- and di-additions of alanine at sn-2 and sn-3 of the glycerol [35,36].

### 4.2. Lipopeptides

Lipotripeptide (L3P: FA-Phe-N-methylVal-Ala-O-methyl) and lipopentapeptide (L5P; FA-Phe-N-methylVal-Ile-Phe-Ala-O-methyl) are cell envelope lipopeptides in *Bacillus subtilis* for which non-ribosomal peptide synthetases (NRPSs: encoded by mps1 gene) assemble the peptide moiety [37,38]. Lipoheptapeptides (2-HydroxyFA-Leu-Ser-Leu-Ile-Thr-Ile-Phe) have also been described for *Rhodococcus equi* [39].

### 4.3. Modified Diacylglycerols

Trihexosyl diacylglyceriols (triHDGs) and acyl-triHDGs are lipids unique to some *Clostridium* spp. and *Romboutia* spp. and are useful biomarkers to identify bacterial subspecies [40,41]. Another unique modified DG family in these bacterial species is N-acetylglucosaminyl-DGs [40,42]. These lipids are also further modified by the addition of a phosphoethanolamine group to the carbohydrate moiety [42].

### 4.4. Siolipin A

**Figure 14:**
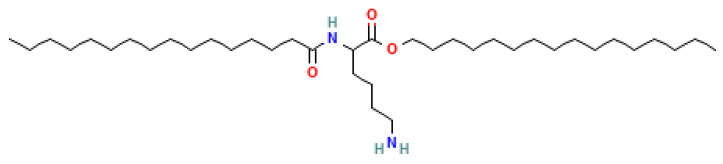
Structure of siolipin A.

The novel lipoamino acid, siolipin A (Figure 14), along with analogs possessing hydroxy fatty acids, is a biomarker for a number of *Streptomyces* spp. [43,44,45].

### 4.5. Quorum Sensing (QS) Molecules: Oligopeptides

**Figure 15:**
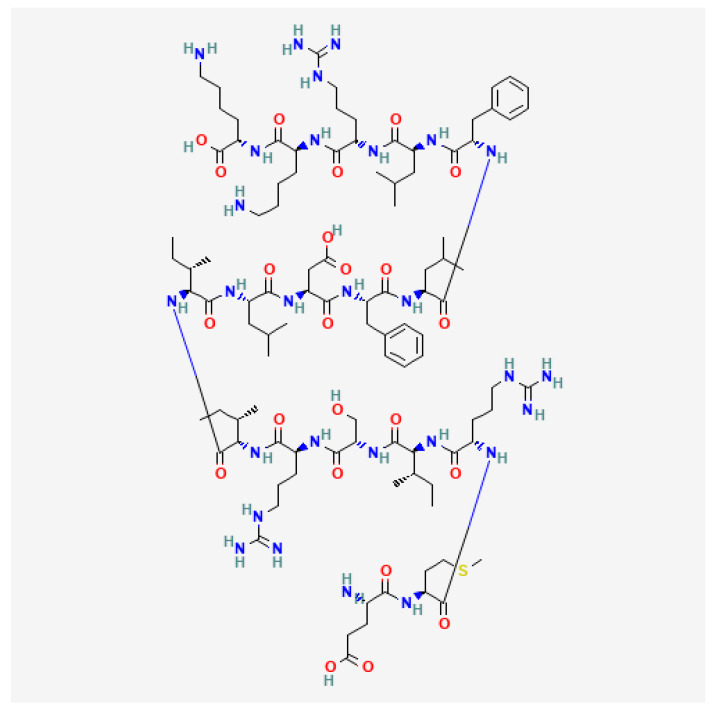
Structure of competence-stimulating peptide-2 (CSP-2), a Gram-positive QS molecule that activates membrane histidine kinase [47].

Gram-positive bacteria utilize oligopeptides (Figure 15) as QS mediators [46]. QS involves cell-to-cell communication, thereby alerting bacteria to potential environmental changes. Gram-negative bacteria utilize N-acyl homoserine lactones as QS mediators ([47]; Section 5.5). In both cases, QS molecules are critically involved in biofilm development, which often results in pathogenicity [47,48].

## 5. Gram-Negative Bacteria

### 5.1. Aminoacyl Fatty Acyls of Hydroxy Fatty Acids (FAHFAs)

Gram-negative bacteria lack the cell wall characteristic of Gram-positive bacteria. Lipid A is a major membrane lipid in the cell envelope, composed of an inner and outer membrane with an intermediate peptidoglycan layer (e.g., *Bacteroiddetes* spp. and *Porphyromonas gingivalis*). Intact lipid A molecules are large and tethered to the membrane, requiring acid hydrolysis prior to mass spectrometric analyses [49].

**Figure 16:**
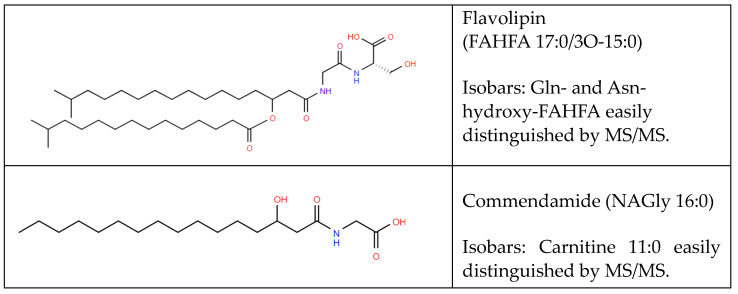
Structure of Gly-Ser-FAHFA 15:0/3-O-17:0, also termed flavolipin and N-(3-Hydroxyhexadecanoyl) glycine (Gly-HFA 16:0) also termed commendamide.

By contrast, a number of lipid A precursors are readily analyzed via conventional lipid extraction procedures. Modified fatty acyls of hydroxy fatty acids (FAHFAs) are one example of these lipid A constituents that are absent from Gram-positive bacteria [50,51,52]. Examples of this are the family of lipodipeptides, Gly-Ser-FAHFA ([51,52,53,54]; Figure 16), the Gly-Ser-FAHFA precursor/metabolite Gly-Ser-HFAs [3,52,53], and the lipotripeptide family, Gly-Ser-Orn-FAHFA, all of which are 3-HFA in the FAHFAs [53]. The lipodipeptides all generate the MS^2^ products Gly, Ser, and Gly-Ser [51]. The 3-HFA substituent of Gly-Ser-3-HFAs is validated with MS^2^ loss of the 3-HFA as an aldehyde, clearly distinguishing from 2-HFAs.

A family of Gly-Ser-FAHFA-phosphatidic acids (Gly-Ser-FAHFA-PAs) has also been isolated from some Gram-negative bacteria (e.g., *P. gingivalis*) [51,55]. These lipopeptides all reside in the outer bacterial membrane [54].

The lipopeptide, Orn-FAHFA family has only been monitored in a number of unique Gram-negative bacterial families, including *Planctomycetes* spp., *Burkholderia* spp., *Agrobacteriumtume faciens*, *Rhizobium* spp., and *α-*, *β-*, and *γ-proteobacteria* [56,57].

### 5.2. Glucosaminylphosphatidylglycerol (GlcN-PG)

**Figure 17:**
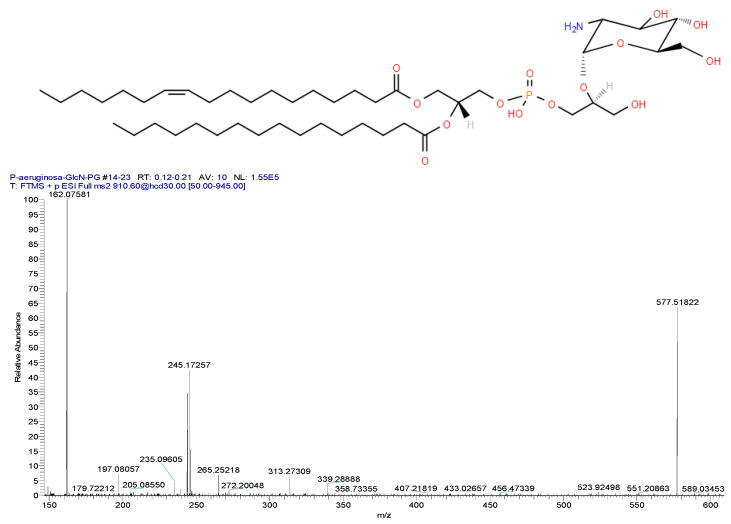
Structure and ESI-HRMS^2^ spectrum of GlcN-PG 34:1 [M + H = 910.6015; 0.89 ppm]^+^ extracted from *P. aeruginosa*. Product ions include [DG 34:1 − H_2_O = 577.5182; 1.4 ppm]^+^, [GlcN − H_2_O = 162.0758; 1.17 ppm]^+^, [FA 18:1 − H_2_O = 265.2522; 1.54 ppm]^+^, [MG 18:1 − H_2_O = 339.2889; 1.38 ppm]^+^, and [MG 16:0 − H_2_O = 313.2731; 1.9 ppm]^+^.

Phosphatidylgycerols (PGs) are dominant glycerophospholipids in the membranes of Gram-negative bacteria and are involved in protein translocation across membranes. *Pseudomonas aeruginosa* modifies these GPLs by glycosylation to generate glucosaminyl-PGs ([58]; Figure 17). These modified PGs may be involved in biofilm formation, which is a major characteristic of *P. aeruginosa* infections. This suggestion is bolstered by recent observations that poly (acetyl,arginyl)glucosamine disrupts biofilm formation by *P. aeruginosa* and resulted in microbial clearance in a rat model of lung infection [59].

Since *P. aeruginosa* infections are associated with high morbidity and mortality; increased understanding of the dynamics and function of GlcN-PGs may be a new approach for the development of antimicrobial therapeutic strategies.

GlcN-PGs appear to be unique to *P. aeruginosa*, in that we have not monitored these lipids in a number of other microbes, presented as follows: *Bacilli *(*+*)*: Bacillus subtilis*, *B. cereus*, *B. megaterium*, *Enterococcus faecalis*, *Staphylococcus epidermis*, *S. mutans*, *S. mitis*, *S. sanguins*, *S. acidominus*, *S. intermedius*, *S. pyogenes*, *S. salivarius*, *S. oralis*; *Actinomyceta *(*+*): *Mycobacterium bovis*, *M. avium*, *M. smegmatis*, *Rhodococcus equi*, *A. viscosus, Micrococcus luteus*, *Corynebacterium glutamicum*; *Bacteriodia *(*-*): *P. gingivalis*, *Prevotella brevis*; *Fusobacteria *(*-*): *Fusobacterium nucleatum*; *Spirochaetia *(*-*): *Treponema denticola*, *Leptospira interrogans*; *Clostridia *(*-*): *Veillonella parvula*; *Verucomicrobiae *(*-*): *Akkermansia muciniphila*; *Gammaproteobacteria *(*-*): *Moraxella bovis*, *Proteus mirabilis*, *P. vulgaris*, *E. coli*, *Moraxella bovoculi*, *Pseudomonas fragi*, *Vibrio vulnificus*; *Alphaproteobacteria *(*-*): *Rickettsia monacensis*; *Campylobacterales *(*-*): *Helicobacter pylori*; *Flavobacteriia *(*-*): *Flavobacterium* spp.; *Halobacteria *(*-*): *Haloferax volcanii*; *Rickettsiales* (*-*): *Rickettsia monacensis*; *Saccharimonadia *(*-*): *Nanobacter lyticus*; and *Fungi*: *C. albicans*, *A. niger*.

### 5.3. Ethanolamine and Glycerol Phosphoryl Ceramides

**Figure 18:**
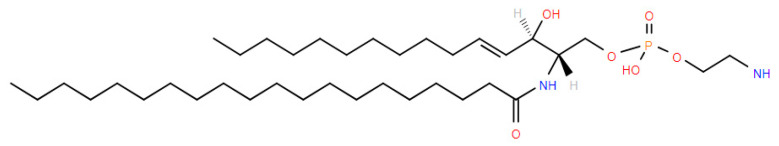
Structure of Ceramide (d15:1/20:0)-PE (CerPE 35:1;O2). Isobars of Cer-PE 35:1;O2 are sphingomelin 32:1;O2 and ceramide aminoethylphosphonate 35:1;O3. These can all clearly be distinguished by MS/MS analysis.

Ethanolamine phosphoryl ceramides [EPCs; Figure 18] and glycerol phosphoryl ceramides (GPCs) are abundant membrane lipids in Gram-negative bacteria (*P. gingivalis*, *Tannerella forsythia*, *Bacteroides fragilis*, *B. thetaiotaomicron*, *Prevotella brevis*, *Proteus vulgaris*, *Fusobacterium nucleatum*, *Veillonella parvula*, *Treponema denticola*, and *Alkermansia muciniphila*) [38,51,60,61]. Another very unique modified ceramide lipid family only reported for Gram-negative bacteria [38] is glycerol bisphosphoceramides (Cer-PGP-Cer). These lipid families are conjectured to be virulence factors and mask bacteria against the host immune response [38].

### 5.4. Hexosyl Ceramides

**Figure 19:**
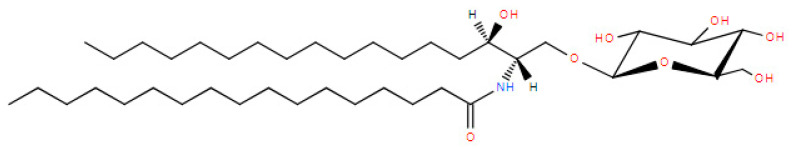
Structure of glucosyl ceramide (d17:0/17:0). In *B. fragilis*, the n-acyl fatty acid is hydroxylated at the β-carbon.

Studies of different strains of *Bacteroides* have demonstrated that only *B. fragilis* possesses hexosyl ceramides [62,63]. The ceramides are d17:0, d18:0, and d19:0, and the fatty acid is a 3-hydroxy fatty acid [Figure 19]. As with Gly-Ser-3-HFA (Section 5.1), the 3-HFA is eliminated from these hexosyl ceramides as an aldehyde with MS^2^. Hexosyl ceramides have also been reported for human and mouse urinary bladders [64] and for sponges [64]. However, these are d18:1, t18:0, and t20 ceramides in urinary bladders and t17:0 ceramides with 2-hydroxy additions to the N-acyl fatty acid in sponges.

### 5.5. Ceramide Sulfonates: Sulfobactins

**Figure 20:**
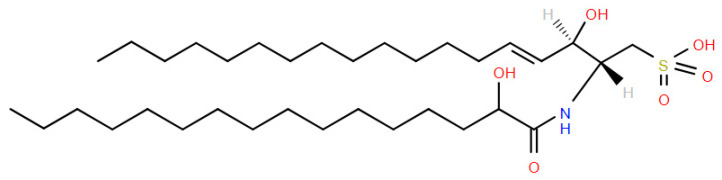
Structure of sulfobacin A. Flavocristamide A, with 2 methy substituents on the fatty acid chains, is an isobar. Chromatography is needed to distinguish these two isobars.

A unique family of sphingolipid sulfonates found in *Capnocytophaga*, *Cytophaga*, *Flexibacter*, *Sporocytophaga*, *Alistipes*, and *Odoribacter* are ceramide sulfonates, which are synthesized in Gram-negative bacteria via N-acylation of capnine instead of sphinganine [65,66]. Structurally, the terminal -CH_2_-OH group of ceramides is replaced by -CH_2_-SO_3_H (Figure 20).

### 5.6. Cholesteryl Acyl-ᾳ-Glycosides (CAGs)

**Figure 21:**
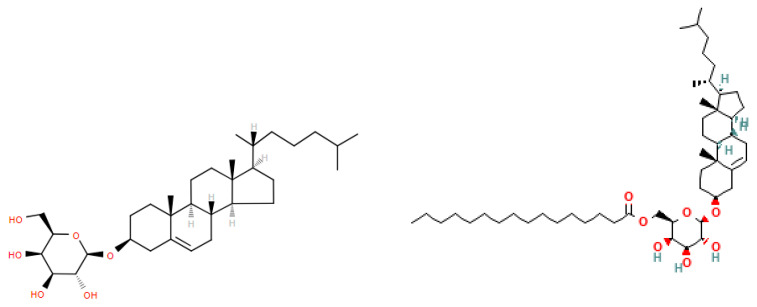
Structures of cholesteryl galactoside and acyl(16:0) cholesteryl galactoside.

CAGs are extremely hydrophobic cholesterol esters (Figure 21) synthesized by a number of Gram-negative bacteria including *Helicobacter pylori* and *Borrelia burgdorferi* [67,68,69,70]. These membrane lipids protect against host lysomal activity [71].

### 5.7. Quorum Sensing (QS) Molecules: N-Acyl Homoserine Lactones (AHL)

**Figure 22:**
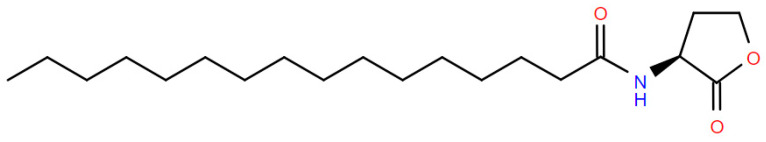
Structure of palmitoyl homoserine lactone (AHL 16:0). Isobars of HSL 16:0 include N-acylethanolamine (NAE) 18:2;O and N-acyl-glycine (NAGly) 18:1, which can be distinguished by MS/MS.

**Figure 23:**
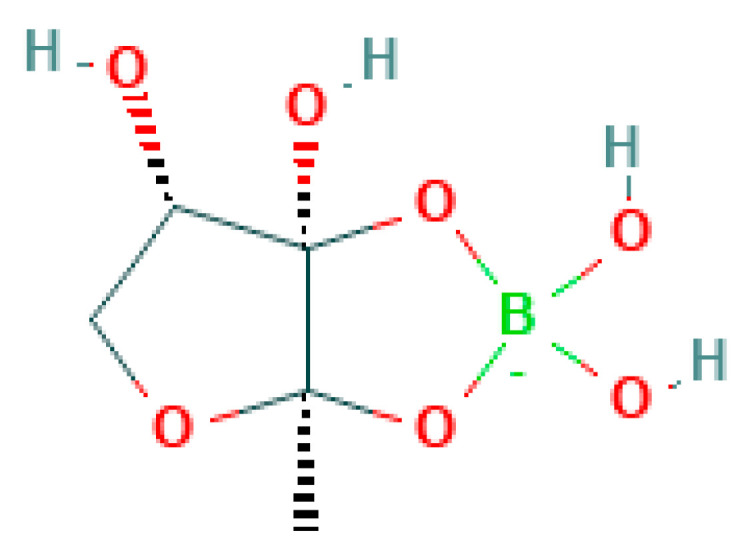
Structure of autoinducer-2 produced by *E. coli* and *Vibrio harveyi*.

Acylhomoserine lactones (Figure 22) are utilized by Gram-negative bacteria for intercellular communication [72,73]. These diffusible signal molecules are essential for bacteria to regulate their population density and associated biofilm formation (e.g., *Pseudomonas aeruginosa*). Interestingly, *Escherichia* spp. and *Salmonella* spp. can sense AHLs produced by other bacterial species but do not synthesize them [74]. These bacteria produce alternate QS molecules like autoinducer-2 ([75]; Figure 23).

## 6. Conclusions

This paper presents a high-level review of unique microbial biomarkers that are useful in microbiological research. Increasing our knowledge base in this area will lead to the improved identification of microbial infections, increased understanding of the complex roles of microbial lipids and secondary metabolites in cellular function and pathogenicity, and the development of new antimicrobial therapies. The importance of HRMS relates to the significant number of lipid and metabolite isobars. Utilizing ESI-HRMS, investigators will be able to evaluate all of the described lipids with a single organic extract [76]. The more polar metabolites can be analyzed utilizing extraction with acetonitrile/methanol/formic acid [77]. These analytical approaches enable the acquisition of a broad range of lipid and metabolite information with two analytical platforms.

## Figures and Tables

**Table 1:** Antifungal cyclic peptide families monitored in cyanobacteria [13,14,15,16,17,18,19,20].

Large Member Families	Small Member Families
Anabaeopeptins (Figure 2) Nodulapeptins Microcystains Cylindrocyclophanes	Brunsvicamides Namalides Brunsvicamides Lyngbaureidamides Ferintoic acids Schizopeptin Pompanopeptin Oscillamides Hapalosins Desmamides

## Abbreviations

AHL, N-Acyl homoserine lactone; d18:0, dihydroxy sphingolipid base; CAG, cholesteryl acyl-ᾳ-glycoside; Cer, ceramide; DGCC, diacylglyceryl carboxyhydroxymethylcholine; DG, diacylglycerol; DGTA, diacylglycerol-hydroxymethyl-trimethyl-alanine; DHDG, dihexosyldiacylglycerol; EPC, ethanolamine phosphorylceramide; ESI-HRMS, electrospray ionization high-resolution mass spectrometry; FA, fatty acid; FAAL, fatty acyl AMP ligase; FAHFAs, Fatty Acyls of Hydroxy Fatty Acids; GIPC, Glycosylinositol-phosphorylceramide; GlcN, glucosamine; GPC, glycerol phosphoryl ceramide; GPL, glycerophospholipid; GroP; glycerol phosphate; h, hydroxy; HFA, hydroxy fatty acid; LTAP, lipoteichoic acid primer; NAE, N-acyl ethanolamide; NAGLY, N-acyl glycine; MG, monoacylglycerol; MGCC, carboxyhydroxymethylcholine; MHDG, monohexosyldiacylglycerol; NRPS, non-ribosomal peptide synthetase; PA, phosphatidic acid; PG, phosphatidylgycerol; PGP, glycerol-1,3-diphosphate; PKS, polyketide synthase; QS, Quorum Sensing; SQDG, sulfoquinovosyldiacylglycerol; SQMG, sulfoquinovosylmonoacylglycerol; t18:0, trihydroxy sphingolipid base; TMM, trehalose monomycolate; triHDG, trihexosyl diacylglyceriol; VLCFA, very-long-chain fatty acid.
